# Understanding Stress: Characteristics and Caveats

**Published:** 1999

**Authors:** Hymie Anisman, Zul Merali

**Affiliations:** Hymie Anisman, Ph.D., is a professor at the Institute of Neuroscience, Carleton University, Ottawa, Canada. Zul Merali, Ph.D., is a professor at the School of Psychology and Department of Cellular and Molecular Medicine, University of Ottawa, Canada

**Keywords:** psychological stress, physiological stress, sensory stimuli, conditioned response, unconditioned response, coping skills, neurotransmitters, brain, neurochemistry, biological adaptation, animal model, genetics and heredity, gender differences, age differences, life event, AODD (AOD use disorders), literature review

## Abstract

Exposure to stressful situations is among the most common human experiences. These types of situations can range from unexpected calamities to routine daily annoyances. In response to stressors, a series of behavioral, neurochemical, and immunological changes occur that ought to serve in an adaptive capacity. However, if those systems become overly taxed, the organism may become vulnerable to pathology. Likewise, the biological changes, if sufficiently sustained, may themselves adversely affect the organism’s well-being. Several factors may dictate an individual’s response to environmental stressors, including characteristics of the stressor (i.e., type of stressor and its controllability, predictability, and chronicity); biological factors (i.e., age, gender, and genetics); and the subject’s previous stressor history and early life experiences. Research on the physiological and psychological responses to different types of stressful stimuli is presented, focusing particularly on processes that may be relevant to the development of alcohol use disorders. Stressful events may profoundly influence the use of alcohol or other drugs (AODs). For example, the resumption of AOD use after a lengthy period of abstinence may reflect a person’s attempt to self-medicate to attenuate the adverse psychological consequences of stressors (e.g., anxiety). Alternatively, stress may increase the reinforcing effects of AODs.

Exposure to stressful situations is among the most common human experiences. These types of situations can range from unexpected calamities (e.g., bereavement, natural disaster, or illness) to routine daily annoyances. Regardless of their degree of severity, however, stressors may promote physiological and behavioral disturbances, ranging from psychiatric disorders (Brown 1993) to immune system dysfunction (Herbert and Cohen 1993). Stressful events also may profoundly influence the use of alcohol or other drugs (AODs). For example, the resumption of AOD use after a lengthy period of abstinence may reflect a person’s attempt to self-medicate to attenuate the adverse psychological consequences of stressors (e.g., anxiety). Alternatively, stress may increase the reinforcing effects of AODs.

This article provides a working definition of stress and describes research on the physiological and psychological responses to different types of stressful stimuli, focusing particularly on processes that may be relevant to the development of alcohol use disorders.

## Stress: A Working Definition

As commonly used, the term “stressor” indicates a situation or event *appraised* as being aversive in that it elicits a stress response which taxes a person’s physiological or psychological resources as well as possibly provokes a subjective state of physical or mental tension. As relevant scientific data have accumulated, however, a simple, universally accepted definition of stress has become increasingly elusive.

This article focuses on some of the factors that may influence the mechanisms by which a person responds to stressful situations (i.e., stressors). Much of the information presented here is based on animal research, which can provide essential information not obtainable from human studies. However, the human stress response is influenced by a host of personality characteristics and life experiences that cannot be duplicated in animal studies. Other articles in this issue provide more specific information on possible interactions between stress and human behavioral responses, such as alcohol consumption.

Many researchers view the stress response as an adaptive mechanism designed to maintain the relative stability of the body’s overall physiological functioning (i.e., homeostasis) in response to a challenge. However, not all stress responses are clearly adaptive. Some physiological reactions to stress that appear to confer short-term benefits are followed by adverse long-term repercussions. In other instances, changes that appear to have adverse consequences may, on closer examination, turn out to be beneficial. Finally, some changes that may have little positive value and no adaptive significance may yet comprise part of the overall stress response.

The ambiguity of the stress response can be illustrated by examining the functions of cortisol, a hormone released by the adrenal glands in response to stressful stimuli (see sidebar, page 247). Among other functions, cortisol helps promote the release of energy stores essential for coping with stress. Yet, cortisol may suppress the normal functioning of the immune system, a response that could theoretically render the body more susceptible to infectious diseases. However, cortisol-induced immune suppression also may serve a protective function (Munck et al. 1984), preventing the development of illnesses characterized by immune attack on the body’s own tissues (e.g., rheumatoid arthritis). Even when cortisol release has adaptive consequences, the elevated cortisol levels persist for an extended period, then the adaptive nature of the response may be lost and adverse effects may ensue. Thus, what we consider to be an adaptive short-term response may subsequently provoke long-term pathophysiological consequences (Sapolsky et al., 1986).

**Regulating the Stress Response**

The maintenance of a relatively stable balance of physiological functions (i.e., homeostasis) is constantly challenged by illness; injury; hostile environmental conditions; unpleasant emotional states; and even certain normal functions, such as sexual activity and exposure to new environments. The body’s response to such stressors is regulated largely by interactions among the hypothalamus, pituitary gland, and adrenal glands, together termed the HPA axis (see figure). In response to potentially harmful stimuli, the hypothalamus, which is located near the base of the brain, secretes two hormones that travel directly to the adjacent pituitary gland. These two hormones, corticotropin-releasing hormone (CRH) and arginine vasopressin (AVP), 1 promote the secretion of adrenocorticotropic hormone (ACTH) from the pituitary gland. Traveling through the bloodstream, ACTH reaches the adrenal glands, which are located on top of the kidneys. In humans, the adrenal glands respond to ACTH by releasing the steroid hormone cortisol into the bloodstream.^2^ Cortisol exerts widespread physiological effects throughout the body, acting in concert with other chemical messengers to help direct oxygen and nutrients to the stressed body site and suppress the immune response, while influencing certain functions, such as appetite and satiety; arousal, vigilance, and attention; and mood.

**Figure:**
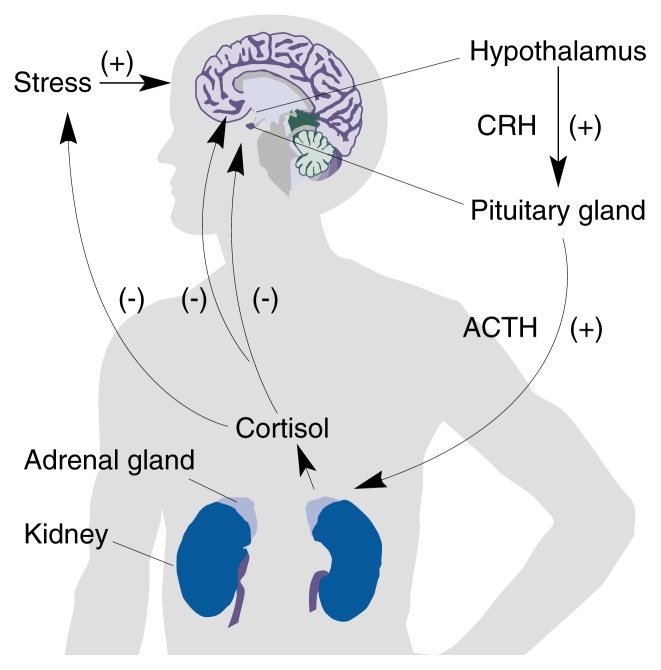
Regulation of the stress response by the hypothalamus-pituitary-adrenal (HPA) axis. ACTH = adrenocorticotropic hormone; CRH = corticotropin-releasing hormone; + = stimulates; − = inhibits.

Under normal circumstances, the presence of cortisol in the bloodstream signals the hypothalamus to terminate CRH secretion, thereby preventing overactivity of the stress response. The regulation of a physiological response through inhibition mediated by the end-product of the response is called negative feedback. When negative feedback control of the HPA axis does not operate adequately, as may occur following chronic stress or as a consequence of certain psychiatric disorders (possibly including severe depression), persistent activation of the HPA axis may occur. Damage resulting from HPA overactivity may include suppression of growth, immune system dysfunction, and localized brain cell damage that might result in impairment of learning and memory.

—Hymie Anisman and Zul Merali

1AVP also serves a key function in maintaining the body’s water balance.2The corresponding hormone in rodents is corticosterone.

Because stressful stimuli often elicit cortisol secretion, some researchers have proposed the use of cortisol levels as an index of the stress response. However, not all events perceived as stressful lead to the release of hormones specifically associated with stress. Indeed, several other hormones and similar chemical messengers are extremely responsive to stressful stimuli and may influence the cascade of events activated by stress. Furthermore, positive stimuli may elicit physiological responses comparable in many respects to those provoked by adverse events, and increased cortisol release is not uniquely provoked by events perceived as stressful. For example, rats that were offered food in the laboratory exhibited activation of the hypothalamic-pituitary-adrenal axis (HPA) (see sidebar) identical to that elicited by stressful stimuli, such as physical restraint (Merali et al. 1998). HPA activation could arguably represent an anticipatory response to any strong stimulus, preparing the animal to respond appropriately. Alternatively, the presentation of food, at least in animals, may actually threaten to disrupt homeostasis. In that case, the stress response may help mobilize the body’s physiological response to the potential onslaught of nutrients, which require digestion and absorption. In addition, food may naturally contain or be contaminated by any number of toxic compounds that must be eliminated or destroyed (e.g., by immune system activity or enzymatic degradation in the liver). Furthermore, in the wild, an animal approaching a food source may experience some risk from either predators or competitors. The evaluation of these hypotheses is complicated by individual differences in the perception and appraisal of a stimulus as stressful.

## Characteristics of the Stressor

Several factors serve a fundamental role in determining the nature and consequences of the stress response (see sidebar). These factors include inherent features of a given type of stressor as well as the conditions under which the stressor is encountered (i.e., the stressor regimen).

### Evaluating the Stress Response

In general, stressors may be psychogenic and/or neurogenic. Psychogenic stressors are purely of psychological origin (e.g., anticipating an adverse event, experiencing the death of a loved one, or caring for a chronically ill person). Neurogenic stressors involve a physical stimulus (e.g., a headache, bodily injury, or recovery from surgery).

In addition, environmental stressors can be classified as either processive or systemic. Processive stressors are those that require appraisal of a situation or involve high-level cognitive processing of incoming sensory information. Examples of processive stressors among animals include exposure to new environments, predators, or situations that trigger fear because of previous association with unpleasant stimuli (i.e., fear cues). In contrast, systemic stressors are of physiological origin (e.g., disturbances of normal bodily metabolism resulting from bacterial or viral infection).

Herman and Cullinan (1997) have suggested that both processive and systemic stressors might activate the HPA axis through distinct but converging neurological circuits. Specifically, processive stressors may primarily activate the limbic system, a region of the brain comprising interconnected structures that are associated with arousal, emotion, and goal-directed behavior. Conversely, systemic stressors may more directly influence the hypothalamus, a brain structure with multiple regulatory functions that interacts extensively with the limbic system. In the absence of experimental evidence, it seems reasonable to speculate that processive stressors might be more closely associated with increased alcohol consumption than would systemic stressors.

When evaluating the impact of adverse events on an individual, a researcher or health professional must consider the specific nature of the stressor involved. Although most stressors elicit some common neurochemical and behavioral effects, their responses are not always identical.

In animal studies, researchers have employed a wide range of stressors to assess behavioral and biological outcomes. Some of these stressors are ethologically sound (i.e., they represent situations that the animal would ordinarily encounter in its natural environment and for which it may have developed natural, evolutionary defenses). Ethological stressors may include the sight or odor of predators, confrontation with unfamiliar members of the same species, or fear cues.

Other commonly employed experimental stressors include exposure to cold air, immersion in cold water, and mild electric shocks administered to the animal’s foot or tail. In various studies, investigators have administered footshock and tailshock at varying intensities, thereby obtaining information on the effects of controllable (i.e., escapable) versus uncontrollable (i.e., inescapable) stressors. However, the generalizability of experimental results involving some stressors is limited. For example, the effects of exposure to cold air or cold water may reflect physiological processes specific to the generation of body heat rather than the psychological consequences of stress.

The nature of the stress response varies depending on the nature of the stressor and the stressor regimen. This fact is illustrated by the phenomenon of adaptation (i.e., a diminished response after prolonged or repeated exposure to a stressor). For example, within a single experimental session, the brain’s chemical response exhibits adaptation to some stressors (e.g., restraint) but is less likely to occur in response to others (e.g., footshock or tailshock). Although the reason for this finding is unknown, one possibility is that restraint is continuous, whereas footshock is intermittent. Thus, drawing firm conclusions about the adverse effects of a stressor based on a specific stressor regimen can sometimes be difficult. The variability of the stress response may provide important clues to the identification of the psychological and physical processes that govern voluntary alcohol consumption.

Stressor effects in humans are more complex than in animals. Some investigations of the human stress response have been conducted under contrived conditions in the laboratory, and the meaningfulness of such studies may be limited. Studies that attempt to simulate natural conditions are more likely to produce realistic outcomes. Some of the latter studies rely on a person’s recollection of past events (i.e., retrospective studies). The disadvantage of retrospective studies, however, includes the potential distortion of recall resulting from subsequent experience or the subject’s current mental state. Prospective studies, which are less commonly employed, involve an initial baseline examination of the subject with subsequent followup evaluations (Sklar and Anisman 1981).

Irrespective of the experimental approach, research clearly indicates that stressors, which are usually multidimensional, produce not only immediate actions but also protracted effects secondary to the primary stressor. For instance, stressful experiences are often followed by persistent brooding (i.e., rumination) that may in itself be stressful, and some events (e.g., bereavement) may have secondary effects (e.g., financial burden and loss of social support). Whereas some stressor effects may diminish over time (e.g., sadness, remorse, or guilt), the effects of other stressors may increase (e.g., financial burden and loss of social support). In addition, the stress response itself may function as a stressor. For example, symptoms of depression induced by stress may lead to interpersonal conflict or, conversely, social withdrawal, further exacerbating depression (Hammen 1991).

With respect to behavioral outcomes, some stressors (e.g., loss of social support) are more likely than others to provoke depressive symptoms (Monroe and Simons 1991), whereas other stressors (e.g., threats or impending stress) are more closely associated with anxiety symptoms (Finlay-Jones and Brown 1981). Surprisingly, stress-induced psychiatric pathology is often elicited not by a major adverse life event but by a series of relatively mild stressors (i.e., day-to-day hassles). Furthermore, the effects of the minor stressors may be especially profound if they occur following a major stressful event (Lazarus 1990; Ravindran et al. 1997).

The severity of stress-induced effects may be related to characteristics of the individual coupled with the nature of the stressor. Relevant stressor characteristics include the following: (1) the degree to which stress can be mitigated or eliminated by an appropriate response (i.e., controllability), (2) the predictability of onset of the stressor, (3) the duration or chronicity of exposure (i.e., either acute or over a relatively protracted period), and (4) the timing and frequency of exposure (e.g., intermittent).

### Controllability and Coping

Perceived controllability clearly influences some (but not all) stress responses. For example, uncontrollable stressors provoke behavioral disturbances in animals that are not induced by controllable stressors of comparable severity. Some investigators interpret these differences as the consequences of “ learned helplessness” (Seligman 1975). Other researchers interpret these findings in terms of the strain that such events place on the neurotransmitter systems in the brain (see textbox, p. 244) (Anisman et al. 1991; Weiss and Simson 1985).

**Nerve Cell Communication and the Stress Response**

Nerve cells communicate with one another through chemical messengers called neurotransmitters. The neurotransmitters discussed in this article interact extensively to perform a variety of regulatory activities. Serotonin affects a wide range of physiological functions, including appetite, sleep, and body temperature. Serotonin also influences emotional states, and its dysfunction has been implicated in both psychiatric and addictive disorders. Dopamine helps regulate goal-directed behaviors (including the reinforcing effects of alcohol and other drugs) as well as certain motor functions. Within the brain, norepinephrine plays a role in arousal and in the modulation of other neurotransmitter systems. When released into the bloodstream by the adrenal glands, norepinephrine functions as a stress-related hormone, preparing the body for “fight or flight” in response to threatening situations.

The excessive strain on, or the resulting variations of, neurotransmitters may increase an individual’s vulnerability to pathological states. Indeed, studies in rodents have indicated that in some brain regions (e.g., the hypothalamus), the response of the neurotransmitter norepinephrine (NE) to uncontrollable stressors is more profound than that provoked by controllable stressors. Likewise, the controllability of stressors may differentially influence the functions of the neurotransmitters serotonin (5-HT) and dopamine (DA) in specific brain regions. In addition, some behavioral disturbances evoked by uncontrollable stressors can be mimicked by drugs that disrupt the functioning of these neurotransmitters. Conversely, treatments that attenuate the neurochemical alterations elicited by stressors limit such behavioral disturbances (Anisman et al. 1991). In effect, an individual’s response to a stressor may be dictated by the availability of appropriate coping strategies, and certain behavioral disturbances may be most pronounced under conditions where stressor controllability is not possible or where coping responses are ineffective.

Although researchers may be tempted to conclude that the ability to neutralize a stressor is the fundamental feature in predicting neurochemical and behavioral change, this conclusion may be premature. For instance, when stressed animals are permitted to fight with a member of their species, the effects ordinarily elicited by uncontrollable stressors may be mitigated, a phenomenon known as displacement (Anisman et al. 1991). Nevertheless, displacement aggression may not eliminate the stressor and may in fact create additional stress. An important aspect of displacement behaviors, such as aggression, is that by offsetting the impact of stressors, the displacement behaviors may become reinforced. AOD use may serve, in part, as such a displacement behavior. Whether or not the displacement behaviors related to stressful events actually support both the initiation and maintenance of AOD abuse remains to be determined.

Not all neurochemical or physiological processes are differentially influenced by stressor controllability. The ability to respond rapidly to a stressful challenge may have greater adaptive value than the ability to assess controllability. Moreover, determining whether a given stressor is controllable may require sustained or repeated exposure, a luxury that may not be affordable. Thus, systems designed for immediate response (e.g., activation of the HPA axis or the immune system) ought to react comparably to both controllable and uncontrollable stressors. Conversely, systems that are uniquely involved in the appraisal of processive stressors might react differently to controllable than to uncontrollable stressors.

Studies in humans support the view that stressor controllability may be fundamental in determining the stress response, despite the fact that in a great number of instances, *control* is actually illusory. Rather than assessing stressor controllability, researchers may find it more profitable to consider the specific coping mechanisms that are available to the individual (Lazarus 1993). Broadly speaking, coping can be subdivided into several subtypes, including emotion-focused coping (e.g., emotional expression, emotional containment, blame, avoidance, denial, and passivity); problem-focused coping; social support; cognitive restructuring; and problem-solving.

Researchers often assume that emotion-focused coping is a relatively ineffective strategy, whereas social buffering, problem-solving, and cognitive restructuring may be more efficacious. To some extent, this conclusion is based on findings that depressed patients, relative to control subjects, tend to favor emotion-focused coping and revert to a more problem-focused strategy with successful treatment (Ravindran et al. 1997). Although emotion-focused coping can be ineffective and even counterproductive, the effectiveness of a strategy may depend on the specific stressor regimen. A given strategy may be ineffective under one set of conditions but be highly effective under another. Ultimately, the abilities to maintain flexibility and be prepared to use different strategies may be the hallmark of effective coping.

### Chronicity and Predictability

Intuitively, one would suspect that the behavioral and neurochemical impact of an acute stressor would be exacerbated by repeated exposure to the stressor. However, some stressor-induced behavioral, neurochemical, and immunological disturbances in rats and mice may be mitigated by prolonged stressor exposure. For example, the decline of brain NE concentrations associated with acute stressor exposure may reverse following protracted or repeated exposure (Weiss and Simson 1985). Such adaptation appears to represent an active process, because NE levels in chronically stressed animals do not simply return to prestress levels but, instead, exceed basal values. Chronic stressors appear to promote a compensatory increase in the production of NE (or, in the case of DA, moderation of excessive utilization), leading to increased neurotransmitter concentrations.

Factors that prevent or limit neuro-chemical adaptation may be associated with behavioral or physiological disorders. For example, some of the behavioral and neurochemical changes associated with *chronic predictable* stressors are less apt to appear following *chronic unpredictable* stressors (Anisman et al. 1991). Interestingly, a regimen of chronic mild stressors may result in an inability to experience pleasure (i.e., anhedonia) similar to that elicited by relatively intense stressors. Thus, even stressors that have modest effects when applied acutely may have pronounced behavioral repercussions when experienced on a chronic, unpredictable basis (Willner 1997).

In humans, stressors are typically of a varied nature, are encountered on an intermittent and unpredictable basis, and may be experienced over protracted periods. As indicated earlier, many stressors have secondary effects (e.g., rumination, financial loss, or loss of social support), which are themselves stressful or limit coping abilities. A chronic, intermittent stressor regimen is less likely to lead to neurochemical adaptation and, hence, favor the development of pathology. When the chronic stressor regimen is not only unpredictable, but is also uncontrollable and associated with secondary stressors, the occurrence of behavioral disturbances might, perhaps, increase (Anisman et al. 1991).

Two important caveats must be stressed with respect to the impact of chronic stressors. First, the compensatory neurotransmitter changes associated with repeated stressor exposure vary widely and occur in several brain regions. Not all of these variations necessarily progress at comparable rates or in all species of laboratory animals. Thus, the nature of the pathology associated with a chronic stressor regimen may depend on the specific neurochemical disturbances incurred. Second, the process of coping with chronic stressor exposure creates prolonged and intense demands on neurochemical systems, a condition termed “allostatic load.” Sustained and excessive allostatic load may culminate in pathological outcomes (Schulkin et al. 1998). Evaluating the contribution of stressors to behavioral disturbances (e.g., alcoholism) in humans requires large-scale prospective studies assessing the impact of acute and chronic insults, the contribution of coping factors, and allostatic load associated with certain stressor regimens.

## Effects of Genetics, Gender, Age, and Previous Stressor Exposure

### Genetic Differences

Both the psychological and physiological responses to a given stressor may vary greatly between individuals, thereby influencing the type of pathology to which a person is vulnerable. Such vulnerability may be influenced by genetic factors.

In mice and rats, behavioral, hormonal, immunological, and neurochemical effects of a given environmental stressor may differ significantly between different genetic strains. For example, some rodent strains exposed to a stressor may display marked HPA alterations or variations of brain neurotransmitter levels, whereas other strains may display fewer or less profound effects. Similarly, the same stressful event may induce opposite effects on certain aspects of immune functioning in different rodent strains. Rather than regarding such interindividual or interstrain variations as a “noise factor,” the experimenter can use them to help identify both the factors that predict the response to a stressor and the occurrence of a pathological state related to the stressor (Anisman et al. 1991, 1998).

Individual or genetic differences in the stress response may indicate either an overall increase of reactivity or a highly specific increase in the reactivity of a particular biological system. Similarly, alterations of transmitter function in one brain region, or alterations of one aspect of immune functioning, do not suggest similar alterations in other brain regions or in other aspects of immunity. Interindividual differences in the fragility of different biological systems may determine why a stressor increases the vulnerability to a particular pathology in one individual but a different pathology in another individual. In addition, if the organism is endowed with increased vulnerability to stressor effects on neurochemical processes as well as increased genetic vulnerability to a particular pathology, then the stressor would be expected to increase the risk for this particular pathology. In the case of alcoholism, genetic factors favoring increased alcohol intake, coupled with an inherited disposition toward excessive stressor reactivity or inappropriate coping styles, could potentially contribute to alcohol abuse.

### Gender

Data concerning gender-dependent effects of stressors are relatively limited, although researchers have found that the HPA response to stressors is greater in female rats than in male rats. This effect appears to occur at almost every level of HPA functioning, and the responses, to some extent, are regulated by interaction among the hypothalamus, pituitary gland, and gonadal organs (Ferrini et al. 1997; Viau and Meaney 1991). Such factors may contribute to the gender differences often seen with respect to some behavioral disturbances (e.g., mood disorders), but the contribution of these factors to AOD consumption is not yet clear.

### Age

In humans, the age-dependent effects of stressors intertwine with numerous psychosocial factors (e.g., reduced physical abilities; financial constraints; and loss of coping resources, social support, and psychological flexibility). Animal studies further suggest that certain neurochemical systems that are sensitive to stressors react differently in aged compared with young individuals. In aged rats, stressor-provoked neurochemical alterations are induced more readily than in young rats, and the return to basal levels of neuronal functioning requires a relatively sustained period of time. Theoretically, stressors should generate rapid neurochemical responses that readily normalize upon stressor termination. Thus, the sustained neuronal activation of aged animals may reflect a lack of adaptability of functioning. Aged animals might therefore be more vulnerable to stressor-provoked pathology (Anisman et al. 1991; Sapolsky et al. 1986). In humans, where aging is frequently associated with reduced coping abilities or opportunities (owing, for example, to diminished social supports following loss of friends and loved ones, reduced physical abilities, and possibly financial concerns), the effects of stressors on pathological processes may be particularly marked. Given that developmental, social, and cultural factors influence not only stressor perception but also individual coping styles (Aldwin 1994). Ultimately, such variables probably contribute to pathological states and should be considered in relating stress to alcoholism.

Similar to an aged animal, however, a very young organism may lack or may not have developed the behavioral and neurochemical repertoire to cope with stressors effectively and thus may be at increased risk for pathology. As discussed shortly, stressors in young animals may act to program (or reprogram) neuronal functioning to increase vulnerability to neurochemical disturbances encountered later in life.

### Effects of Prior Life Events or Stressor Exposure

#### Sensitization

Stressful events not only have marked immediate effects but also may influence one’s response to later stressor experiences. Such a sensitization effect may be responsible for the high rates of relapse associated with psychiatric disorders, such as depression (Post 1992). Studies in animals have indicated that exposure to stressors typically induces physiological changes that persist for a relatively brief duration. However, if animals are reexposed to the same stressor at a later time, then the neurochemical changes in the brain occur more readily. Such effects have been noted with respect to several neurotransmitters, but particular attention has been devoted to the analysis of norepinephrine and dopamine (see reviews in Kalivas and Stewart 1991). Interestingly, these effects have not only been observed when the reexposure session involves the same stressor, but also when it involves an entirely different stressor (Nisenbaum et al. 1991). Furthermore, such cross-sensitization effects have been witnessed between processive stressors and drug treatments. Thus, treatment with amphetamine or cocaine may enhance the response introduced by subsequent exposure to a stressor (Kalivas and Stewart 1991).

The sensitization appears to depend on the characteristics of the stressors to which the animal had previously been exposed. As indicated earlier, young animals that have been exposed to an acute stressor demonstrate increased activity of NE and DA when they are later exposed to a stressor. As a result, reexposure may result in declining neurotransmitter levels. However, in animals that have been exposed to a chronic stressor regimen, subsequent reexposure to the stressor induces a sensitization with respect to both synthesis and utilization of NE and DA. As a result, the level of the neurotransmitter does not decline readily. In effect, the nature of the previous stressor experiences (and the neurochemical changes engendered) determine an animal’s response to stressor reexposure and thus might also influence behavioral responses engendered by subsequent challenges (Anisman et al. 1991).

When animals are repeatedly exposed to a particular stressor, adaptation may occur and, consequently, neurotransmitter alterations may become progressively less pronounced. However, when animals are subsequently introduced to a stressor not previously encountered, then the adaptation is not evident, and a marked neurochemical change is again elicited. Thus, the adaptation that occurs with sustained exposure to a stressor may be unique to that particular stressful stimulus, and diminished responsivity may not occur in response to a new stimulus. Conversely, a chronic stressor regimen may result in an increased response following exposure to a different type of stressor. In effect, it seems that although repeated exposure to a particular stressor may promote either adaptation with respect to stressor appraisal or some aspects of neuronal functioning (e.g., the stressor is appraised as being less aversive, or variations occur with respect to either the receptor sensitivity and/or number present at presynaptic or postsynaptic sites, or with respect to transmitter release), these processes may be affected in a different fashion when a novel stressor is introduced, culminating in augmented neuronal functioning.

Studies by Tilders and colleagues (Tilders and Schmidt 1998; Tilders et al. 1993) have revealed important processes concerning the sensitization of neuroendocrine functioning that occur in response to both processive and systemic stressors. These investigators have found that stressors may induce prolonged changes of neuroendocrine functioning within certain neurons of the hypothalamus that communicate with the pituitary gland. As discussed in the sidebar, both corticotropin-releasing hormone (CRH) and arginine vasopressin (AVP) can stimulate the release of adrenocorticotropic hormone (ACTH) and, hence, corticosterone release from the adrenal glands. Furthermore, AVP may potentiate the effects ordinarily elicited by CRH. With the passage of time following stressor exposure, the CRH neurons may co-produce AVP, thus rendering the HPA axis more sensitive to stressors. Essential features of these findings include the following: (1) changes of AVP and CRH co-production may be long lasting and thus account for some of the protracted effects of stressors that have been reported, and (2) the long-term effects of stressors also could be provoked by the administration of cytokines (i.e., substances that act as signaling molecules within the immune system), suggesting that immune activation also may proactively influence the response to subsequently encountered adverse experiences.

#### Early Life Stimulation

The stimulation or handling of laboratory animals during their first few weeks after birth (which also entailed a brief separation from their mothers) was found to decrease age-related learning disturbances and increased resistance to the effects of later stressors (Meaney et al. 1996). Animals that had experienced stimulation during the first 21 days of life showed basal concentrations of ACTH and corticosterone comparable to that of nonstimulated animals. However, as adults, when exposed to a stressor, the stimulated animals displayed blunted ACTH and corticosterone responses and a faster return to basal hormone levels. These long-lasting variations may have involved a cascade of neuronal changes, culminating not only in altered regulatory processes associated with HPA functioning (Meaney et al. 1996) but also in variations with respect to the propensity to consume alcohol during later adulthood (Lancaster 1998; Jones et al. 1985).

Liu and colleagues (1997) conducted studies to determine why brief handling involving separation from the mother (i.e., for as little as 15 minutes per day) had such pronounced and persistent effects. After reuniting with their young following the brief separation, mothers exhibited increased licking, grooming, and nursing of their offspring. Moreover, because the high levels of these maternal responses were correlated with altered hormonal responses to stressors, the researchers suggested that maternal behavioral style acted to “program” HPA responses to later environmental stressors. Whether such factors also contribute to alcohol intake remains to be established.

Anisman and colleagues (1998) studied two mouse strains that exhibit very different behavioral and neurochemical profiles in response to stressors. The more stress-reactive strain displayed relatively poor maternal behavior, spent less time within the nest, and took longer to retrieve young offspring, which had been placed in different portions of the cage, compared with the less stress-reactive strain (Anisman et al. 1998). Thus, the exaggerated response to stressors in the more reactive mice may be related in part to maternal factors. When young mice of the stress-reactive strain were raised by mothers from the less reactive strain (cross-fostered on the day of birth), some behavioral disturbances and the exaggerated HPA alterations of the more reactive mice were decreased. However, maternal behavior alone is not sufficient for this outcome to emerge. In particular, being raised by a mother from the more reactive strain did not engender behavioral or hormonal disturbances in young mice of the more resilient strain. This finding implies that heightened stress reactivity in these mice results from a combination of genetic factors and inadequate maternal care (Zaharia et al. 1996).

#### Early Life Deprivation

In contrast to early life stimulation, early life stressors (e.g., separation from the mother for relatively long periods, such as 3 hours per day) may increase the potential for later stressor-promoted HPA activation. Indeed, protracted separation provokes an increase of plasma ACTH and corticosterone and increased behavioral and neuroendocrine reactivity to stressors encountered during adulthood (Meaney et al. 1996). Paralleling the effects of early life deprivation—in which young animals were made ill by the administration of a bacterial toxin—several aspects of the HPA response to stressors during adulthood were increased (Shanks et al. 1995). Of course, bacterial toxins may induce fever, possibly altering the mother’s behavior toward the young (e.g., elevations of body temperature may serve as a cue for termination of nursing), which, in turn, precipitates the altered response to subsequently encountered stressors. It remains to be determined whether metabolic stressors that do not elevate body temperature also induce such long-term effects. In any case, early life trauma, which includes not only separation from the mother but also bacterial infection, appears to have potentially far-reaching implications. Thus, various early life experiences in newborn humans might significantly affect reactivity to stressors encountered during adulthood.

## Summary

In response to stressors, a series of behavioral, neurochemical, and immunological changes occur that ought to serve in an adaptive capacity. However, if these systems become overly taxed, the organism may become vulnerable to pathology. Likewise, the biological changes, if sufficiently sustained, may themselves adversely affect the organism’s well-being. Several factors may dictate an individual’s response to environmental stressors, including characteristics of the stressor (i.e., type of stressor and its controllability, predictability, and chronicity); biological factors (i.e., age, gender, and genetics); and the subject’s previous stressor history and early life experiences. Ultimately, these factors interact to determine the organism’s biological responses to environmental stressors; thus, not surprisingly, much interindividual variability exists with respect to the impact of stressors. Of course, the retinue of biological changes and the broad range of variables that influence these outcomes often make it difficult to identify the mechanisms associated with stressor-provoked pathology.
